# Seasonal Variation in Nutritional Value and Technical Quality of Lionfish (*Pterois miles*) from the Ionian and Aegean Seas

**DOI:** 10.3390/foods14132353

**Published:** 2025-07-02

**Authors:** Mado Kotsiri, Dimitra Kogiannou, Chrisanthi Nikoloudaki, Ioannis Kleidas, Aikaterini Dogrammatzi, Paraskevi K. Karachle, Kriton Grigorakis

**Affiliations:** 1 Institute of Marine Biology Biotechnology and Aquaculture (IMBBC), Hellenic Centre for Marine Research (HCMR), 190 13 Anavyssos, Greece; dkogiannou@hcmr.gr (D.K.); cnikol@hcmr.gr (C.N.); giannis_kl@hotmail.com (I.K.); 2 Institute of Marine Biological Resources and Inland Waters (IMBRIW), Hellenic Centre for Marine Research (HCMR), 164 52 Argyroupoli, Greece; dogramatzi@hcmr.gr (A.D.); pkarachle@hcmr.gr (P.K.K.)

**Keywords:** Mediterranean, fatty acids, composition, invasive species, quality

## Abstract

Lionfish (*Pterois miles*), an invasive species in the Mediterranean, pose ecological and socioeconomic challenges. This study examines the seasonal variation in the nutritional composition and technical quality of lionfish from the Ionian and Aegean Seas, evaluating their potential as a food resource. Fillets were high in protein (19.4%) and low in fat (2.0%), with significant seasonal differences in the Ionian Sea, where winter samples had higher lipid content. The fillet yield (28.4%) was satisfactory given the fish’s morphology. Fatty acid analysis confirmed lionfish as a valuable source of polyunsaturated fatty acids (PUFAs), particularly eicosapentaenoic acid (EPA) and docosahexaenoic acid (DHA), with EPA + DHA levels exceeding the recommended daily intake (119.2%). Seasonal variations in fatty acid composition were observed, including higher EPA in autumn and lower lipid nutritional quality in summer. Arachidonic acid (ARA) was also present at nutritionally significant levels (79.4 mg/100 g). The n3/n6 ratio (2.2) and favourable atherogenic and thrombogenic indices highlight its nutritional benefits. This is the first study to assess seasonal variations in the nutritional value and technical yield of lionfish in the Mediterranean, offering novel insights into its commercial valorisation. These findings support its promotion as a sustainable protein source and as a means of managing its invasive population.

## 1. Introduction

The Indo-Pacific Devil firefish, commonly known as the lionfish *Pterois miles* (Bennett, 1828), is considered one of the most ecologically disruptive invasive species in marine ecosystems. This invasion poses a severe threat to marine biodiversity, ecosystem structure and functioning through altering trophic dynamics and adversely affecting native biota. As a result, it can have socioeconomic impacts, affecting fisheries and, consequently, local economies [1,2,3,4]. Lionfish are characterised by high reproductive rates, rapid growth and broad diet flexibility, which, along with the absence of natural predators in newly invaded ecosystems, give them a competitive advantage over native species [1,5,6,7].

Lionfish invasion in the Caribbean Sea, which began in the early 2000s, is one of the most well-documented cases and demonstrates the species’ capacity to spread rapidly and have a negative impact on native biodiversity. The feeding efficiency of lionfish has led to severe population declines in ecologically important species, including herbivorous fish. The decline of these herbivores has led to increased algal overgrowth on coral reefs, which, in turn, reduces coral recruitment and resilience [2,7,8,9]. Efforts to mitigate lionfish invasion in the Caribbean have included community-based lionfish culling programmes, promoting the fish as a culinary delicacy, and exploring economic incentives to support lionfish fisheries [10,11]. While these methods seem promising, the rapid reproduction rate and large geographic range of lionfish make eradication unlikely, underscoring the need for continued management.

In the Mediterranean, lionfish were first recorded in Israel in 1991 [12]. Following a period without any additional records, the invasion started in 2012 [13,14], as habitat suitability in the region and increasing water temperatures due to climate change have facilitated the spread of this species throughout the region, particularly in the warm, oligotrophic waters of the eastern Mediterranean. Since its initial sight in the eastern Mediterranean, the species has spread across various regions, reaching the Aegean Sea by 2009 and later expanding to the Ionian Sea by 2018 [15]. Like in the Caribbean, lionfish in the Mediterranean pose a risk to fisheries by threatening native fish communities, disrupting food web dynamics and competing with commercially valuable species [16]. In addition, their venomous spines can pose a risk to human health and, potentially, affect coastal activities [3,4,17,18].

Invasive species management is challenging in marine environments, but recent research suggests that promoting the commercial exploitation of lionfish could help reduce its population growth, turning an environmental problem into an economic opportunity. By marketing lionfish as an environmentally friendly seafood choice, there is potential to reduce its ecological impact while enriching the Mediterranean seafood market [11,19].

Studies on the biochemical composition, such as protein and lipid content, as well as the broader technical and biochemical quality of lionfish are essential for its consumption and certification of its suitability as a food source [20,21,22]. Despite the presence of systematic research that focuses on its ecological impact and management strategies, as mentioned above, there is data scarcity with respect to eating quality, namely, the composition and technical yields of this species. Therefore, the aim of this study was to assess the technical quality and nutritional value of lionfish from the Ionian and Aegean Seas seasonally, addressing a gap in the literature and, potentially, providing insights into sustainable management strategies.

## 2. Materials and Methods

### 2.1. Sampling, Somatometric Indices and Proximate Composition

**Figure 1:**
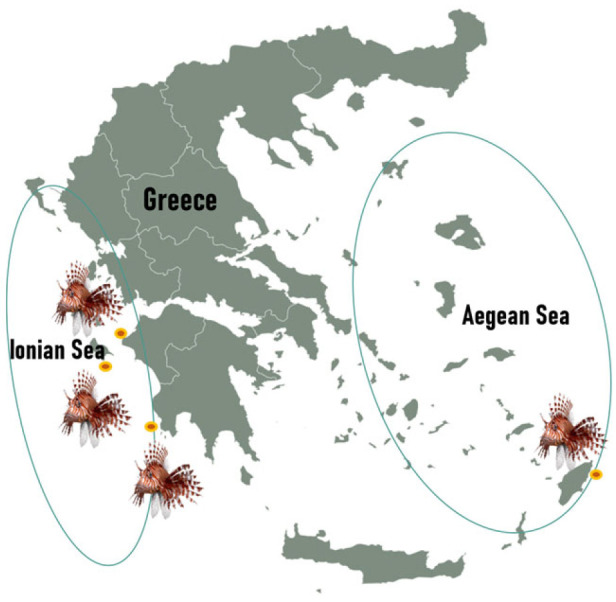
Short map of Greece and geographical sampling distribution.

The specimens were caught by professional, small-scale fishermen using standard fishing methods and handling procedures. The lionfish were frozen immediately after being caught and delivered to HCMR, where they were stored at −20 °C. Samples from the Ionian Sea were collected from Zakynthos Island and Pylos (Messinia, West Peloponnese Peninsula), while samples from the Aegean Sea were collected exclusively from Rhodes Island (Figure 1). Sampling was conducted on a seasonal basis over a one-year period, with specimens collected in autumn (October–November), winter (January–February), spring (April–May) and summer (July–August). These timeframes were chosen to reflect the typical seasonal cycles of the eastern Mediterranean region.

The frozen samples were gradually brought to 0–3 °C in a controlled cold room for a short time (a few hours) to allow further processing and simultaneously avoid bio-chemical tissue alterations. At this temperature, the fish remained partially frozen, which helped preserve tissue integrity and allowed for more precise and consistent filleting. Morphological characteristics, such as body length (total and standard; TL and SL, respectively, in mm), body weight (total and gutted, in g) and total visceral fat (in g), were subsequently measured, and each individual was also sexed.

A total of 31 individuals were collected from the Ionian Sea (18 females and 13 males), with an average weight of 261.4 ± 150.6 g, and 79 individuals were collected from the Aegean Sea (47 females and 32 males), with an average weight of 241.1 ± 99.3 g. The fish were then filleted, lyophilised and stored frozen for further analysis. Somatic measurements were used to determine technical quality, including condition index (CI), dressing yield (DY), filleting yield (FY) and visceral fat index (VFI). For these estimations, lionfish were manually scaled, gutted and filleted with precision to maximise muscle recovery from the flank. Each somatometric index was calculated individually according to the following formula, providing a comprehensive assessment of the morphological and yield-related characteristics of the fish:

(1) CI=[100 × (body weight/body length)]

(2) DY=[100 × (gutted body weight/body weight)]

(3) FY=[100 × (fillet weight/body weight)] 

(4) VFI=[100 × (total visceral fat weight/body weight)] 

The proximate composition of lionfish fillets was analysed via standard AOAC procedures (2005) [23]. The moisture content was determined by drying the samples at 105 °C until a constant weight was reached, whereas the ash content was measured after incineration at 500 °C for 16 h. The crude protein content was estimated by determining the nitrogen content via the Kjeldahl method after acid digestion, using the conversion factor % N × 6.25. The total fat content was quantified through petroleum-ether extraction via the Soxhlet method.

The number of individuals analysed for somatometric indices and proximate composition are presented in Supplementary Tables S1 and S2.

### 2.2. Fatty Acid Composition and Nutritional Indices

The fatty acid composition of lionfish fillets was determined by analysing fatty acid methyl esters (FAME) after extraction and direct transesterification using a methanol-toluene and acetyl chloride-methanol solution, as described by Lepage and Roy (1984) [24] with modifications Alexi et al. (2019) [25]. FAME were analysed via gas chromatography–mass spectrometry (7890A GC system, Agilent Technologies, Santa Clara, CA, USA) equipped with a DB–WAX column (Agilent Technologies, Santa Clara, CA, USA). Fatty acid identification was performed by comparison of mass spectra with reference compounds and the NIST spectral library (NIST/EPA/NIH Mass Spectral Library with Search Program, software version 2.0f). Analyses of fatty acid composition and nutritional indices were carried out on six individuals per season at each location (Aegean–Ionian), and the results are expressed as mg/100 g fillet. The fatty acid profile data were then used to calculate lipid nutritional quality indices. The indices were calculated as follows.

The atherogenic index (AI) and thrombogenic index (TI) were estimated according to Ulbricht and Southgate (1991) [26], using the following equations:

(5) $$\mathrm{AI}=[C12:0+\left(4\;\times C14:0\right)+C16:0]/\Sigma \mathrm{UFA}\;$$

and

(6) $$\mathrm{TI}=\left(C14:0+C16:0+C18:0\right)/\left[\begin{array}{l} \left(0.5\;\times \Sigma \mathrm{MUFA}\right)+\left(0.5\;\times \Sigma n6\;\mathrm{PUFA}\right)+\;\;\\ \left(3\times \Sigma n3\;\mathrm{PUFA}\right)+\left(n3/n6\right) \end{array}\right]$$

The hypocholesterolemic/hypercholesterolemic ratio (HH) was calculated according to Santos-Silva et al. (2002) [27], using the following equation:

(7) HH=(cis-C18:1+ΣPUFA)/(C14:0+C16:0)

### 2.3. Statistical Analysis

A multiple linear regression model was used to assess significant differences among season, sex and location and to calculate the percentage of the total variation in the studied parameters explained by these three individual factors and their combined effects. To this end, a general linear model analysis was adopted to compute tests between-subjects effects for all factors, as well as for their combinations. The percentage of total variation explained by each factor or combination can then be calculated by dividing the Type III sum of squares by the corrected total sum of squares. T-test was used to assess the differences in the means of each factor between the two sexes and, similarly, between the two locations. One-way analysis of variance (ANOVA) was used to compare seasonals differences in the means of each factor, followed by Tukey’s multiple comparison test after verification of the normality of the data (Kolmogorov–Smirnov; Shapiro–Wilk test). The results are expressed as the means ± standard deviations (st. dev.). The results were considered significant when *p* < 0.05, and analysis was performed using SPSS version 25.0 and GraphPad Prism v.9.

## 3. Results

Table 1 shows the technical yields of lionfish in different seasons and locations. The DY was higher in spring at both locations (*p* < 0.05), whereas the CI, FY and VFI were unaffected by season or location. However, the samples from the Aegean Sea appear to have had a higher total FY than those from the Ionian Sea. In the Aegean Sea, the different seasons did not affect the levels of protein, fat or moisture, whereas the seasonal factor significantly affected the levels of ash in the fillet. In the Ionian Sea, on the other hand, winter was the season of the year where there was a significantly higher fat content in the fillets than there was in spring and summer (Table 2). Overall, sex had no effect on the somatometric indices or proximate composition at either location.

The fatty acid composition, expressed as the content of the major fatty acid groups and the content of the major polyunsaturated fatty acids (PUFAs) in mg/100 g fillet (Table 3 and Table 4), revealed that lionfish are rich in PUFAs. In the Aegean Sea, the overall mean n3/n6 ratio was 2.6; seasonal variations were evident, with a higher value in autumn and a lower value in summer. A similar pattern was also observed for n3 eicosapentaenoic acid (EPA, 20:5n3) (*p* < 0.05), whereas the opposite was observed for the lipid nutritional quality index HH (Table 3). The overall mean n3/n6 ratio in the Ionian Sea was 1.9, which was lower than that in its Aegean Sea counterpart but not significantly different. The level of DHA was significantly higher in autumn and winter and decreased over time (*p* < 0.05) (Table 4). The total content of SFAs, MUFAs, PUFAs and n-3 fatty acids and EPA + DHA was higher in winter than in the other seasons in the Ionian Sea, whereas the HH index differed significantly between winter and spring (*p* < 0.05) (Table 4). Supplementary Figure S1 provides a visual summary of the most relevant seasonal patterns in fatty acid composition in the form of a heat map.

Furthermore, no significant differences in fatty acid composition were observed between the sexes at any of the locations in the Aegean or Ionian Seas (*p* > 0.05). Supplementary Figure S2 shows these results.

**Figure 2:**
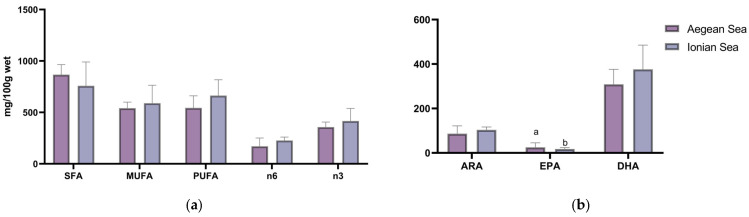
(**a**) Fatty acid composition (mg/100 g fillet): total saturated fatty acids (SFAs), monounsaturated fatty acids (MUFAs), polyunsaturated fatty acids (PUFAs), (**b**) 20:4n6 fatty acids (ARAs), 20:5n3 fatty acids (EPAs) and 22:6n3 fatty acids (DHAs). Letters represent statistically significant differences (*p* < 0.05).

A comparison of the fatty acid compositions at two different locations is shown in Figure 2. Among the measured components, only EPA was found to be significantly higher in the Aegean Sea.

Table 5 summarizes the proportion of the total variation in the measured parameters explained by each factor separately and by their interactions. Location predominantly influenced the lipid nutritional quality indices (AI, TI and HH), while the n3/n6 ratio and ash content were more strongly associated with season. FY variation was also mainly attributed to location. Other factors and interaction effects accounted for smaller proportions of the variation, as shown by the lower values in the Table 5.

## 4. Discussion

The quantity and quality of food consumed by fish are affected by various parameters, including environmental factors, seasonal food preferences, sex, food availability and predator size [28,29,30,31,32,33]. Therefore, regional factors can lead to differences in fish feeding habits and trophic interactions even within the Mediterranean Sea, highlighting the importance of localised research to understand and effectively manage fish populations.

The present study provides a comprehensive analysis of the seasonal variation in the nutritional value and technical quality of lionfish from the Ionian and Aegean Seas. The results of this study confirm that lionfish is a protein-rich species with a low fat content, a characteristic typical of many Mediterranean marine fish [34]. This finding is consistent with previous research by Ayas et al. (2018) [21], who analysed a limited sample of lionfish from the north-eastern Mediterranean and reported similar proximate composition values (moisture, crude protein and total lipids of 75.7–77.6 g/100 g fillet, 20.1–21.1 g/100 g fillet and 1.11–1.84 g/100 g fillet, respectively). These results suggest that lionfish have relatively stable nutritional profiles in different geographical regions of the Mediterranean. From a technical quality perspective, the average fillet yield (FY) of lionfish (28.4 ± 1.4%) is considered satisfactory, given that the species has a relatively large head and frame compared with its muscle mass [35]. This yield makes lionfish a viable option for commercial seafood production, as, on the basis of our present data, it takes 1.3 lionfish to consume a typical portion of 100 g of fillet from an average fish weight of 270 g.

The effects of the spawning season on lipid content and fatty acid composition are well documented in Mediterranean fish species. During reproductive periods, lipid reserves are often mobilised to support gonadal development, resulting in lower total lipid content and variations in fatty acid composition [36,37]. In our study, the effect of seasonality was more pronounced in the Ionian Sea, where the fat content of the winter fillets was higher than that of the spring and summer fillets. Conversely, no significant seasonal differences were observed in the Aegean Sea, suggesting that environmental factors or regional dietary variations may influence lipid metabolism. The higher fat content of winter samples in the Ionian Sea, but not in the Aegean Sea, may be attributed to regional differences in environmental conditions, particularly sea temperature, prey availability and metabolic demands. Lionfish in the Ionian Sea may benefit from greater prey abundance or reduced energy expenditure during winter, enabling greater lipid accumulation. Furthermore, variations in spawning timing and reproductive activity between regions could affect the allocation of energy reserves, with lipid mobilisation occurring differently depending on local ecological conditions. A similar regional and seasonal variation in lipid content and composition has been documented in other Mediterranean fish species [30,36,37], highlighting the influence of localised environmental and biological factors on nutritional profiles. Season was a major determinant of the variation in the n3/n6 ratio, suggesting strong temporal effects on fatty acid composition (Table 5). Notably, EPA levels decreased during the summer months in both regions, supporting the notion that essential fatty acids are allocated to reproductive processes during spawning. The extended spawning season of lionfish in the Mediterranean, occurring mainly in summer but also extending into autumn [38,39], may contribute to the observed stability in total composition and fatty acid content across seasons. Climate change and rising sea temperatures may also play a role in modifying reproductive timing, potentially reducing seasonal fluctuations in nutrient composition.

In terms of nutritional quality, lionfish from the Aegean Sea exhibited a higher nutritional value, with a n3/n6 ratio of 2.6 and an EPA content of 25.3 mg/100 g fillet. The species was also found to be rich in total PUFA content, further emphasising its health benefits. Arachidonic acid (ARA), an essential fatty acid involved in brain function, the immune response and inflammation regulation [40], was present at relatively high concentrations (96.6 ± 24.1 mg/100 g fillet).

The elevated levels of ARA observed in lionfish are due to a combination of dietary, metabolic and ecological factors. Wild lionfish primarily consume teleosts and crustaceans, which often contain high levels of ARA. Comparative studies have demonstrated that wild lionfish exhibit significantly higher ARA concentrations than those in managed environments do, emphasising the importance of natural dietary sources [41]. In the Mediterranean Sea, lionfish exhibit opportunistic feeding behaviour, preying on abundant local species such as small fish and decapod crustaceans. The composition of their prey varies by region (e.g., up to 82.9% fish in the Aegean Sea and up to 95% decapods around Kastellorizo Island) [42], reflecting dietary plasticity and the potential for ARA accumulation through trophic transfer. In addition to their diet, marine fish possess species-specific enzymatic capacities, namely, desaturases and elongases, for the endogenous biosynthesis of long-chain polyunsaturated fatty acids, including ARA. This may further contribute to the observed levels in lionfish tissues [43].

ARA levels in lionfish fillets are nutritionally significant and contribute significantly to the daily dietary intake of this essential fatty acid. While ARA intake from typical dietary sources such as eggs, chickens and fatty fish ranges from 100 to 250 mg/day, a 100 g portion of lionfish provides 96.6 mg, indicating that it is a valuable dietary source. Although this amount is lower than that found in annular seabream (*Diplodus annularis*, 270 mg) and shi drum (*Umbrina cirrose*, 120 mg) [44], it still represents a meaningful contribution to a balanced diet. Notably, in very common species such as wild seabream (*Sparus aurata*) and European seabass (*Dicentrarchus labrax*), ARA levels are lower (30 mg and 20 mg, respectively) than those presently found for lionfish [45].

In addition, the lionfish at both sites presented an optimal n3/n6 ratio of 2.2, much higher than the minimum desired target of 0.5–1, an intake considered essential for maintaining human health and preventing cardiovascular diseases. This ratio highlights the potential of the species as an excellent source of n3 PUFAs. The total EPA + DHA content (0.368 g/100 g fillet) exceeds the FAO recommended daily intake of 0.250 g/day [46], with lionfish providing an EPA + DHA% of the recommended daily intake of 147.2%. This value is comparable to or even higher than that of several commercially important Mediterranean fish, such as common dentex (*Dentex dentex*, 0.27 g/100 g fillet), white grouper (*Epinephelus aeneus,* 0.2 g/100 g fillet), white seabream (*Diplodus sargus*, 0.25 g/100 g fillet) and red seabream (*Pagrus pagrus*, 0.22 g/100 g fillet) [34]. Furthermore, the sum of EPA + DHA in lionfish is close to that reported by Monteiro et al. (2018) [47] for farmed European seabass (*D. labrax*) (0.270–0.480 g/100 g fillet), although the latter has much higher fillet lipid contents.

In terms of cardiovascular health, the AI and TI values further highlight the beneficial fatty acid composition of lionfish. With an AI of 0.68, lionfish fall within the optimal range for fish (0.21–1.41), suggesting its potential role in reducing LDL cholesterol and total cholesterol levels [26,48]. The TI value of 0.49 is also well within the range considered beneficial for cardiovascular health (0.14–0.87) [46], indicating a favourable balance between pro-thrombogenic and anti-thrombogenic fatty acids. Furthermore, the HH ratio of lionfish (1.51) suggests a balanced FA profile, comparable to that of other healthy seafoods, where values typically range from 1.54–4.83 [49]. The nutritional lipid quality indices exhibited the highest proportion of variance explained by location, indicating that geographical differences play a significant role in shaping these lipid-related traits (Table 5).

In addition to their macronutrient profile, lionfish offer other benefits as a seafood option. Previous research has suggested that their muscle tissue contains low levels of heavy metals, ensuring that consumption does not pose a significant risk to human health [21]. Although lionfish is safe to eat, basic safety measures should be taken when handling it, such as wearing puncture-resistant gloves and trimming the spines with scissors or shears, to avoid envenomation before preparation. Given its favourable nutritional profile, high palatability and lack of health risks, promoting lionfish consumption could be a practical strategy to create harvesting pressure, helping to control invasive populations while providing a high-quality seafood option.

Overall, lionfish is a highly nutritious and environmentally friendly seafood option. Owing to their optimal fatty acid composition, including high ARA levels, favourable n3/n6 ratio and significant contributions to daily EPA + DHA intake, lionfish stand out as promising candidates for human consumption. In addition to its nutritional value, the consumption of lionfish offers an ecological advantage. As an invasive species in the Mediterranean, its targeted harvest could contribute to population control while providing an alternative seafood choice. Given their excellent nutritional profile and moderate fat content, lionfish are excellent choices for both dietary diversification and marine conservation efforts.

While the nutritional and ecological benefits of lionfish are evident, developing a successful commercial market also depends on consumer perception, market readiness and stakeholder engagement. Recent studies from the Mediterranean region have highlighted an increase in consumer awareness and acceptance of invasive fish species, including lionfish, particularly when the ecological benefits are communicated effectively [50,51]. However, there are still some barriers in place, such as low familiarity with the species, safety concerns relating to its venomous spines and limited market availability. These factors may reduce consumer willingness to purchase unless supported by targeted education, traceability assurance, and culinary promotion. Moreover, cooperation among stakeholders, including restaurants, suppliers and policymakers, is essential for overcoming entry barriers and creating a stable demand pipeline [52]. Therefore, the broader success of lionfish as a seafood option will require coordinated efforts in public awareness, supply chain development and integration into local gastronomic culture, in addition to demonstrating its nutritional and ecological merits. Future research should continue to explore these socioeconomic dynamics to support marine conservation and the sustainable exploitation of this invasive species.

## Figures and Tables

**Table 1:** Means ± st. dev. of the condition index (CI), dressing yield (DY), filleting yield (FY) and visceral fat index (VFI) in the different seasons for the Aegean and Ionian Seas.

Aegean Sea					
	Autumn	Winter	Spring	Summer	Mean Value
CI	1.39 ± 0.19	-	1.40 ± 0.25	1.40 ± 0.13	1.40 ± 0.00
DY (%)	87.7 ± 3.6 ^a^	-	89.9 ± 4.0 ^b^	86.6 ± 2.9 ^a^	88.1 ± 1.7
FY (%)	30.0 ± 2.6	-	29.7 ± 1.4	29.7 ± 1.9	29.8 ± 0.1
VFI (%)	3.81 ± 1.35		4.28 ± 2.08	4.1 ± 1.07	4.13 ± 0.28
**Ionian Sea**					
CI	1.53 ± 0.17	1.54 ± 0.18	1.41 ± 0.17	1.35 ± 0.24	1.46 ± 0.09
DY (%)	86.8 ± 3.8 ^a^	89.9 ± 1.6 ^ab^	89.6 ± 2.1 ^b^	88.2 ± 2.8 ^ab^	88.6 ± 1.4
FY (%)	28 ± 1	28.1 ± 1.7	27.1 ± 2.2	26.5 ± 1.4	27.4 ± 0.8
VFI (%)	3.87 ± 1.41	5.30 ± 1.03	4.12 ± 1.80	3.49 ± 1.55	4.20 ± 0.78

Letters represent statistically significant differences (*p* < 0.05) between seasons.

**Table 2:** Mean ± st. dev. of fillet proximate composition (g/100 g) by season and sex for the Aegean and Ionian Seas.

Aegean Sea							
	Autumn	Winter	Spring	Summer	Mean Value	Female	Male
Protein	19.6 ± 0.6	-	19.2 ± 0.7	19.4 ± 1.7	19.4 ± 0.2	19.2 ± 0.6	19.6 ± 0.7
Fat	1.70 ± 1.12	-	2.23 ± 1.36	1.95 ± 1.18	1.96 ± 0.27	2.29 ± 0.67	1.63 ± 0.36
Ash	1.37 ± 0.10 ^a^	-	1.20 ± 0.00 ^b^	1.35 ± 0.11 ^a^	1.31 ± 0.09	1.29 ± 0.05	1.35 ± 0.11
Moisture	76.8 ± 1.4	-	77.6 ± 1.7	77.4 ± 1.8	77.3 ± 0.4	77.3 ± 0.5	77.3 ± 1.0
**Ionian Sea**							
Protein	20.0 ± 1.2	19.3 ± 1.2	18.9 ± 1	18.9 ± 1	19.3 ± 0.5	19.4 ± 0.9	19.1 ± 0.8
Fat	2.30 ± 0.35 ^ab^	2.68 ± 0.57 ^a^	1.54 ± 0.46 ^b^	1.55 ± 0.54 ^b^	2.02 ± 0.57	1.89 ± 0.74	2.22 ± 0.37
Ash	1.44 ± 0.11	1.25 ± 0.10	1.40 ± 0.10	1.42 ± 0.10	1.38 ± 0.09	1.37 ± 0.05	1.38 ± 0.11
Moisture	76.5 ± 1.3	77 ± 1.4	78.1 ± 1	77.5 ± 1.1	77.3 ± 0.7	77.2 ± 1.0	77.5 ± 1.2

Letters represent statistically significant differences (*p* < 0.05) between seasons.

**Table 3:** Fatty acid profile (mg/100 g fillet) and lipid nutritional quality indices of lionfish (n = 6/season) from the Aegean Sea in different seasons of the year.

	Autumn	Spring	Summer	Mean Value
12:00	0.5 ± 0.5	0.8 ± 0.5	0.7 ± 0.5	0.7 ± 0.5
14:00	66.2 ± 52.3	90.7 ± 74.6	66.0 ± 52.0	74.3 ± 58.1
14:01	1.2 ± 1.1	1.4 ± 1.1	1.3 ± 1.3	1.3 ± 1.1
15:00	11.3 ± 7.4	18.8 ± 14.7	14.8 ± 10.7	15.0 ± 11.1
16:00	517.9 ± 341.5	628.0 ± 416.1	538.9 ± 342.2	561.6 ± 349.4
16:1n9	12.7 ± 10.1	16.0 ± 10.3	15.3 ± 11.3	14.7 ± 10.0
16:1n7	102.6 ± 74.9	146.3 ± 109.9	113.7 ± 83.6	120.9 ± 87.3
16:1n11	-	5.3 ± 3.2	4.9 ± 2.4	5.1 ± 2.7
17:00	19.1 ± 12.0	27.8 ± 18.0	25.6 ± 16.4	24.1 ± 15.2
17:1n7	6.4 ± 4.2	11.7 ± 7.6	11.2 ± 7.2	9.8 ± 6.6
18:00	160.3 ± 95.1	199.3 ± 111.7	188.2 ± 103.7	182.6 ± 98.9
18:1n9	309.0 ± 213.6	353.0 ± 207.5	304.1 ± 182.0	322.0 ± 190.6
18:1n7	41.0 ± 25.8 ^a^	5.6 ± 3.2 ^b^	5.1 ± 3.2 ^b^	17.2 ± 22.4
18:2n9	9.0 ± 7.5	9.0 ± 4.9	10.0 ± 6.6	9.3 ± 6.0
18:2n6	22.0 ± 14.0	31.6 ± 18.2	25.1 ± 14.9	26.3 ± 15.4
18:3n6	1.3 ± 0.9	3.7 ± 2.3	3.5 ± 2.5	2.8 ± 2.2
18:3n3	4.5 ± 3.1	9.3 ± 6.2	6.7 ± 4.2	6.8 ± 4.9
20:00	5.8 ± 3.6	9.8 ± 5.9	8.3 ± 4.6	8.0 ± 4.9
20:1n11	2.1 ± 1.7	4.7 ± 2.6	6.0 ± 5.7	4.3 ± 3.9
20:1n9	21.8 ± 15.8	45.8 ± 38.7	26.8 ± 22.5	31.5 ± 27.8
20:1n7	2.0 ± 1.4	5.5 ± 3.4	5.0 ± 3.7	4.2 ± 3.2
20:2n9	6.6 ± 8.1	6.3 ± 2.7	7.0 ± 5.2	6.6 ± 5.4
20:2n6	4.3 ± 2.7	9.1 ± 5.2	8.0 ± 4.2	7.1 ± 4.4
20:3n6	2.0 ± 1.2 ^a^	7.2 ± 2.5 ^b^	8.5 ± 4.0 ^b^	5.9 ± 3.9
**20:4n6 (ARA)**	48.0 ± 24.4	94.9 ± 40.9	117.0 ± 76.6	86.6 ± 57.2
20:3n3	1.1 ± 0.7 ^a^	5.5 ± 2.1 ^b^	6.6 ± 2.9 ^b^	4.4 ± 3.1
**20:5n3 (EPA)**	48.4 ± 30.2 ^a^	15.2 ± 10.3 ^b^	12.2 ± 7.2 ^b^	25.3 ± 24.5
22:4n6	2.3 ± 1.4 ^a^	27.6 ± 14.2 ^ab^	36.8 ± 33.3 ^b^	22.2 ± 24.7
22:5n6	2.4 ± 1.8 ^a^	31.7 ± 16.8 ^b^	28.8 ± 16.4 ^b^	21.0 ± 18.6
22:5n3	16.8 ± 10.1	3.0 ± 1.4	15.8 ± 32.8	11.9 ± 19.7
**22:6n3 (DHA)**	246.1 ± 161.6	379.9 ± 228.6	300.7 ± 183.9	308.9 ± 190.2
24:1n9	8.5 ± 3.4 ^a^	27.4 ± 12.5 ^b^	22.3 ± 13.2 ^ab^	19.4 ± 13.0
SFA	783.2 ± 512.5	975.2 ± 640.6	842.5 ± 528.6	867.0 ± 535.7
MUFA	509.7 ± 346.5	609.5 ± 388.1	503.2 ± 315.4	540.8 ± 333.7
PUFA	410.2 ± 258.0	634.0 ± 335.8	586.6 ± 328.3	543.6 ± 307.0
n6	77.7 ± 42.2	205.8 ± 92.2	227.7 ± 142.7	170.4 ± 116.9
n3	316.9 ± 204.9	412.8 ± 248.4	342.0 ± 207.6	357.2 ± 211.9
EPA + DHA	294.5 ± 191.3	395.1 ± 238.9	312.9 ± 190.8	334.1 ± 200.7
n3/n6	4.0 ± 1.1 ^a^	2.0 ± 0.6 ^b^	1.7 ± 0.7 ^b^	2.6 ± 1.3
AI	0.8 ± 0.1	0.8 ± 0.1	0.7 ± 0.1	0.8 ± 0.1
TI	0.6 ± 0.0	0.5 ± 0.1	0.6 ± 0.1	0.6 ± 0.1
HH	1.2 ± 0.1 ^a^	1.3 ± 0.1 ^ab^	1.4 ± 0.2 ^b^	1.3 ± 0.2

Results are expressed as the means ± st. dev. Letters represent significant differences (*p* < 0.05) between seasons. SFA = sum of saturated fatty acids; MUFA = sum of monounsaturated fatty acids; PUFA = sum of polyunsaturated fatty acids; n6 = sum of n6 fatty acids; n3 = sum of n3 fatty acids; EPA + DHA = eicosapentaenoic acid plus docosahexaenoic acid; n3/n6 = sum of n3 and the sum of the n6 ratio; AI = index of atherogenicity; TI = index of thrombogenicity; HH = hypocholesterolemic/hypercholesterolemic ratio.

**Table 4:** Fatty acid profile (mg/100 g fillet) and lipid nutritional quality indices of lionfish (n = 6) from the Ionian Sea in different seasons of the year.

	Autumn	Winter	Spring	Summer	Mean Value
12:00	1.3 ± 0.4	1.1 ± 0.3	0.6 ± 0.1	0.8 ± 0.4	0.9 ± 0.4
14:00	90.2 ± 17.0 ^ab^	119.7 ± 39.5 ^a^	48.7 ± 14.1 ^b^	52.2 ± 24.8 ^b^	74.3 ± 36.6
14:01	1.8 ± 0.5	2.1 ± 1.0	1.1 ± 0.3	1.2 ± 0.6	1.5 ± 0.7
15:00	17.7 ± 4.2	24.0 ± 7.0	10.6 ± 3.9	10.5 ± 4.1	15.0 ± 7.0
16:00	527.8 ± 80.1 ^ab^	630.4 ± 149.9 ^a^	348.7 ± 98.1 ^b^	357.4 ± 135.6 ^b^	452.4 ± 160.0
16:1n9	18.7 ± 3.3	22.1 ± 7.2	13.1 ± 3.6	12.8 ± 5.9	16.2 ± 6.1
16:1n7	161.6 ± 29.6 ^ab^	184.8 ± 62.5 ^a^	91.4 ± 28.5 ^b^	102.2 ± 44.5 ^b^	130.9 ± 55.0
16:1n11	10.7 ± 1.8	12.7 ± 2.8	4.5 ± 1.5	6.1 ± 2.5	8.1 ± 3.9
17:00	25.9 ± 5.9	35.1 ± 6.9	17.4 ± 5.6	17.0 ± 6.4	22.9 ± 9.2
17:1n7	12.9 ± 2.6	16.3 ± 4.1	8.7 ± 3.1	9.1 ± 3.3	11.4 ± 4.3
18:00	188.4 ± 34.6 ^ab^	214.4 ± 50.9 ^a^	128.9 ± 37.8 ^a^	126.5 ± 40.0 ^b^	160.2 ± 53.2
18:1n9	384.1 ± 58.7 ^ab^	443.9 ± 112.6 ^a^	269.4 ± 79.9 ^ab^	259.8 ± 105.0 ^b^	330.1 ± 114.0
18:1n7	8.5 ± 1.4	10.2 ± 2.2	4.4 ± 1.5	5.7 ± 2.4	7.0 ± 2.8
18:2n9	12.8 ± 2.5	14.9 ± 7.6	10.9 ± 3.3	9.1 ± 3.7	11.6 ± 4.6
18:2n6	35.0 ± 7.0	46.5 ± 7.9	20.0 ± 5.3	24.4 ± 9.3	30.4 ± 12.2
18:3n6	4.8 ± 1.2	5.0 ± 1.1	2.9 ± 1.3	3.8 ± 2.2	4.1 ± 1.7
18:3n3	12.8 ± 1.9	16.6 ± 3.9	6.5 ± 1.9	8.9 ± 3.8	10.8 ± 4.7
20:00	10.9 ± 2.7	13.0 ± 3.5	6.8 ± 2.2	7.6 ± 2.6	9.3 ± 3.5
20:1n11	6.8 ± 3.0	6.7 ± 0.5	5.1 ± 2.2	5.1 ± 2.7	5.8 ± 2.4
20:1n9	44.6 ± 9.1	52.0 ± 19.2	23.7 ± 7.5	26.5 ± 10.0	35.4 ± 16.0
20:1n7	9.5 ± 3.4	8.4 ± 1.3	5.3 ± 2.3	6.2 ± 2.8	7.2 ± 3.0
20:2n9	9.3 ± 2.8	10.0 ± 4.5	8.2 ± 2.5	7.2 ± 3.3	8.6 ± 3.2
20:2n6	15.2 ± 3.9	14.6 ± 1.8	7.8 ± 2.3	9.8 ± 4.1	11.6 ± 4.4
20:3n6	14.1 ± 4.2	13.2 ± 2.3	9.1 ± 3.9	9.5 ± 3.4	11.3 ± 4.0
**20:4n6 (ARA)**	122.5 ± 39.5	97.3 ± 25.7	93.6 ± 35.8	102.6 ± 45.1	104.3 ± 36.9
20:3n3	7.9 ± 1.1	8.7 ± 1.9	4.4 ± 1.6	5.2 ± 2.1	6.4 ± 2.4
**20:5n3 (EPA)**	22.3 ± 4.4	23.4 ± 7.5	12.1 ± 3.2	12.6 ± 4.0	17.0 ± 6.9
22:4n6	39.8 ± 15.5	29.7 ± 11.6	30.9 ± 12.5	30.2 ± 14.4	32.7 ± 13.3
22:5n6	34.0 ± 5.6	37.4 ± 5.9	28.1 ± 10.4	27.1 ± 9.1	31.1 ± 8.6
22:5n3	5.9 ± 2.5	5.9 ± 1.3	2.4 ± 0.7	3.7 ± 1.4	4.4 ± 2.1
**22:6n3 (DHA)**	420.9 ± 63.5 ^ab^	508.6 ± 145.1 ^a^	304.1 ± 91.8 ^b^	271.7 ± 61.6 ^b^	364.5 ± 125.8
24:1n9	37.3 ± 6.4	48.7 ± 14.6	22.6 ± 7.0	23.8 ± 6.6	31.9 ± 13.2
SFA	862.2 ± 139.2 ^ab^	1037.6 ± 256.6 ^a^	561.7 ± 159.1 ^b^	572.1 ± 211.7 ^b^	735.1 ± 267.3
MUFA	681.9 ± 110.7 ^ab^	789.5 ± 211.9 ^a^	439.6 ± 130.9 ^b^	448.1 ± 179.5 ^b^	572.7 ± 210.6
PUFA	757.5 ± 117.9 ^ab^	831.7 ± 186.1 ^a^	541.1 ± 160.8 ^ab^	525.8 ± 152.9 ^b^	648.7 ± 194.9
n6	265.5 ± 73.8	243.6 ± 39.1	192.4 ± 68.6	207.4 ± 85.4	225.4 ± 72.0
n3	469.8 ± 70.0 ^ab^	563.2 ± 154.3 ^a^	329.6 ± 98.5 ^b^	302.1 ± 71.8 ^b^	403.1 ± 139.3
EPA + DHA	443.1 ± 66.8 ^ab^	532.0 ± 151.5 ^a^	316.3 ± 94.8 ^b^	284.3 ± 65.3 ^b^	381.5 ± 132.1
n3/n6	1.9 ± 0.5	2.3 ± 0.3	1.8 ± 0.5	1.6 ± 0.4	1.9 ± 0.3
AI	0.6 ± 0.0	0.7 ± 0.1	0.6 ± 0.0	0.6 ± 0.1	0.6 ± 0.1
TI	0.4 ± 0.0	0.4 ± 0.1	0.4 ± 0.0	0.4 ± 0.1	0.4 ± 0.1
HH	1.6 ± 0.1 ^ab^	1.6 ± 0.3 ^a^	1.8 ± 0.0 ^b^	1.7 ± 0.2 ^ab^	1.7 ± 0.2

Results are expressed as the means ± st. dev. Letters stand for statistically significant differences (*p* < 0.05) between seasons. SFA = sum of saturated fatty acids; MUFA = sum of monounsaturated fatty acids; PUFA = sum of polyunsaturated fatty acids; n6 = sum of n6 fatty acids; n3 = sum of n3 fatty acids; EPA + DHA = eicosapentaenoic acid plus docosahexaenoic acid; n3/n6 = sum of n3 and the sum of the n6 ratio; AI = index of atherogenicity; TI = index of thrombogenicity; HH = hypocholesterolemic/hypercholesterolemic ratio.

**Table 5:** Proportion of the total variation (%) explained by factors and their combined effects. The strength of the relationship is visually represented by a colour gradient, where darker shades of green indicate stronger relationships between the factors. CI = condition index; DY = dressing yield; FY = filleting yield; SFA = sum of saturated fatty acids; MUFA = sum of monounsaturated fatty acids; PUFA = sum of polyunsaturated fatty acids; n6 = sum of n6 fatty acids; n3 = sum of n3 fatty acids; ARA = arachidonic acid; EPA = eicosapentaenoic acid; DHA = docosahexaenoic acid; EPA + DHA = eicosapentaenoic acid plus docosahexaenoic acid; n3/n6 = sum of n3 and the sum of the n6 ratio; AI = index of atherogenicity; TI = index of thrombogenicity; HH = hypocholesterolem-ic/hypercholesterolemic ratio.

	Season	Location	Sex	Season * Location	Season * Sex	Location * Sex	Season * Location * Sex
DY	4.7	0.4	11.4	1.8	0.8	0.3	1.2
CI	2.9	3.1	0.8	5.1	1.0	3.6	1.4
FY	9.5	33.1	4.5	1.4	4.8	2.2	5.2
Moisture	13.2	0.3	1.2	1.9	17.3	0.1	0.6
Protein	0.2	0.9	2.5	8.0	17.6	0.3	2.7
Fat	8.7	0.5	0.9	7.6	4.3	8.6	4.8
Ash	30.0	20.4	0.9	5.8	5.7	0.2	0.4
SFA	0.6	17.3	3.4	1.2	4.5	3.5	4.3
MUFA	0.7	7.7	3.5	1.4	4.5	3.6	5.5
PUFA	6.3	2.1	2.2	4.2	5.9	4.5	6.4
n6	22.9	0.0	0.2	10.6	4.1	4.1	4.1
n3	1.7	4.5	3.0	1.8	5.6	3.4	6.1
ARA	22.7	0.2	0.0	5.6	4.1	2.1	3.3
EPA	23.1	12.7	0.0	17.0	2.0	0.0	2.1
DHA	4.2	2.6	3.0	3.8	5.0	3.3	5.0
EPA + DHA	2.5	3.9	2.7	2.1	5.1	3.0	5.3
n3/n6	33.8	14.3	1.4	17.9	0.5	0.0	0.1
AI	16.2	50.6	4.6	2.0	11.4	0.4	2.0
TI	2.3	47.5	1.6	0.6	10.1	0.2	0.5
HH	8.2	60.5	3.9	1.5	12.4	0.8	1.3

## Data Availability

The original contributions presented in the study are included in the article/Supplementary Materials, further inquiries can be directed to the corresponding author.

## Supplementary Materials

The following supporting information can be downloaded at: https://www.mdpi.com/article/10.3390/foods14132353/s1, Table S1. Number of individuals sampled to estimate somatometric indices in the Aegean and Ionian Seas; Table S2. Number of individuals analysed for proximate composition in the Aegean and Io-nian Seas; Figure S1. A visual summary of the most relevant seasonal patterns in fatty acid composition (mg/100 g fillet), presented as a heat map for the Aegean Sea (**a**) and the Ionian Sea (**b**); Figure S2. Fatty acid composition (mg/100 g fillet) in the Aegean and Ionian Seas: (**a**,**b**) Total saturated fatty acids (SFAs), monounsaturated fatty acids (MUFAs), polyunsaturated fatty acids (PUFAs); (**c**,**d**) 20:4n6 (ARA), 20:5n3 (EPA), and 22:6n3 (DHA) fatty acids.
