# Supplementary material for: Seasonal Variation in Nutritional Value and Technical Quality of Lionfish (Pterois miles) from the Ionian and Aegean Seas
Source: Foods. 2025 Jul 2;14(13):2353. doi: 10.3390/foods14132353 (PMC12248952; doi:10.3390/foods14132353)
Supplement: Supplementary file 1 [file foods-14-02353-s001.zip › Supplementary Material.pdf]

Supplementary Material

Table S1. Number of individuals sampled to estimate somatometric indices in the Aegean and Ionian Seas.

	**Aegean Sea**				
	**Autumn**	**Winter**	**Spring**	**Summer**	**Sum**
CI	50	-	10	19	**79**
DY (%)	50	-	10	19	**79**
FY (%)	6	-	6	6	**18**
VFI (%)	49		10	19	**78**
	**Ionian Sea**				
CI	10	2	9	10	**31**
DY (%)	10	2	9	10	**31**
FY (%)	6	2	5	7	**20**
VFI (%)	9	2	9	10	**30**

Table S2. Number of individuals analysed for proximate composition in the Aegean and Ionian Seas.

**Aegean Sea**						
Autumn	Winter	Spring	Summer	Female	Male	Sum
6	-	6	6	9	9	**18**
**Ionian Sea**						
5	2	5	6	10	8	**18**

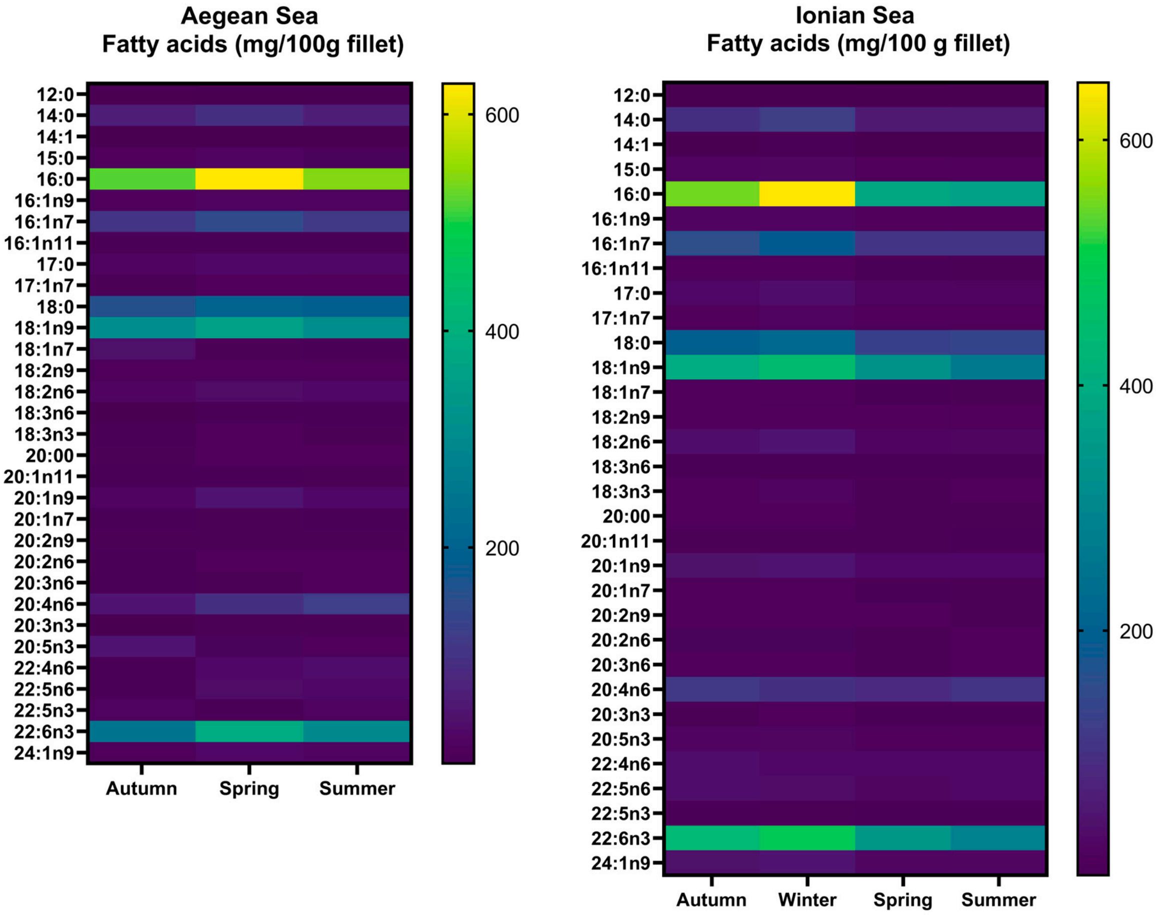

(**a**) (**b**)

Figure S1. A visual summary of the most relevant seasonal patterns in fatty acid composition (mg/100 g fillet), presented as a heat map for the Aegean Sea (**a**) and the Ionian Sea (**b**).

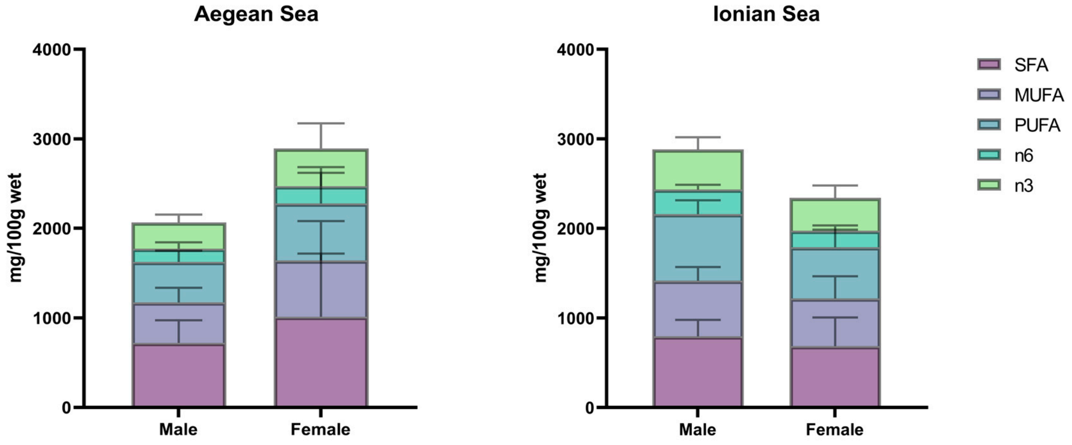

(**a**) (**b**)

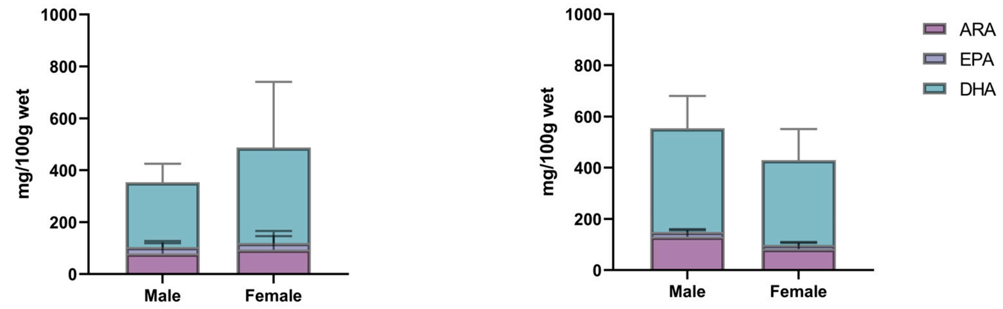

(**c**) (**d**)

Figure S2. Fatty acid composition (mg/100 g fillet) in the Aegean and Ionian Seas: (**a**-**b**) Total saturated fatty acids (SFAs), monounsaturated fatty acids (MUFAs), polyunsaturated fatty acids (PUFAs); (**c**-**d**) 20:4n6 (ARA), 20:5n3 (EPA), and 22:6n3 (DHA) fatty acids.
