# Supplementary figures and images for: Seasonal Variation in Nutritional Value and Technical Quality of Lionfish (Pterois miles) from the Ionian and Aegean Seas
Source: Foods. 2025 Jul 2;14(13):2353. doi: 10.3390/foods14132353 (PMC12248952; doi:10.3390/foods14132353)

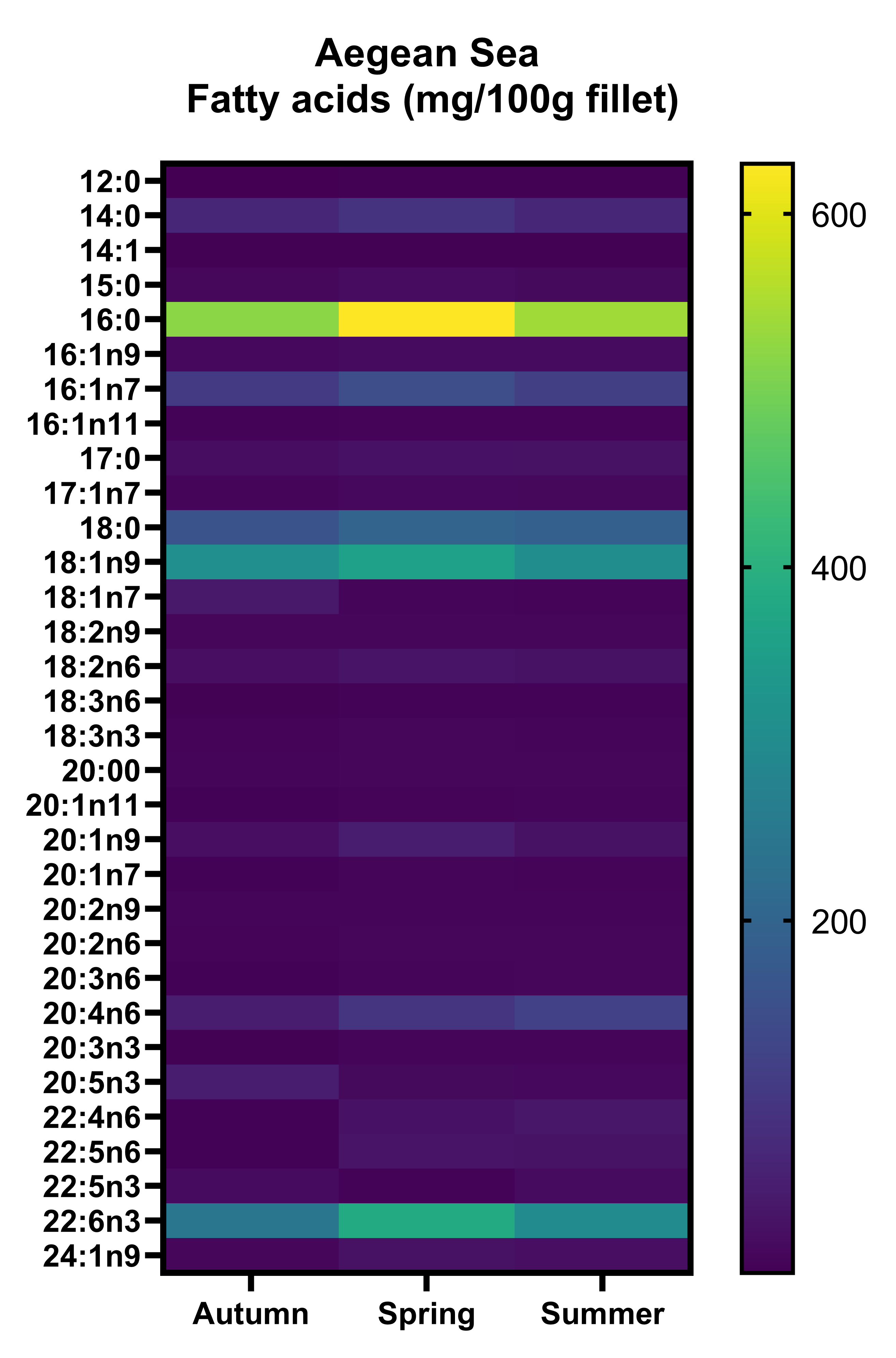

Supplement: Supplementary file 1 [file foods-14-02353-s001.zip › Figure S1a.tif]

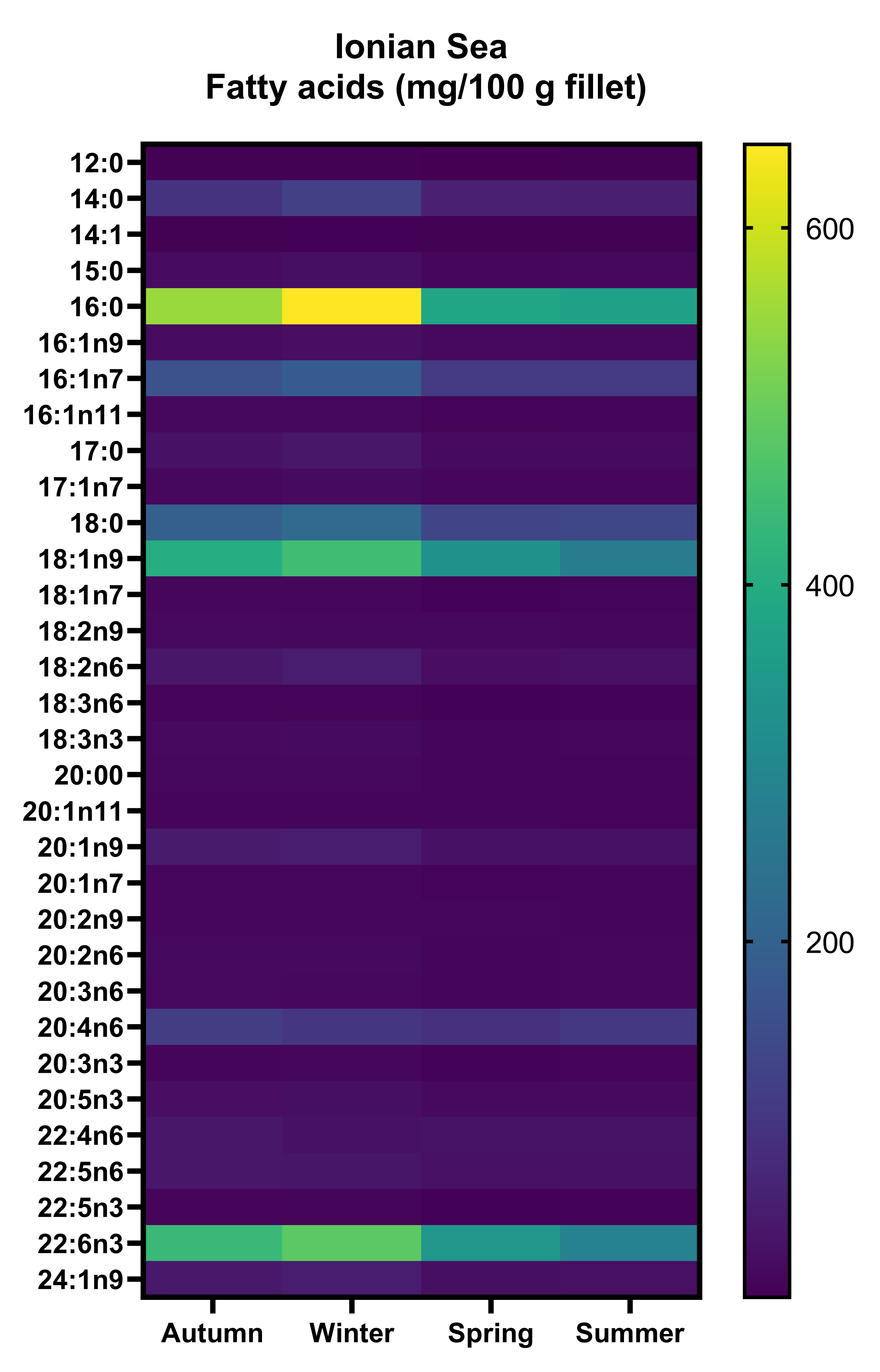

Supplement: Supplementary file 1 [file foods-14-02353-s001.zip › Figure S1b.tif]

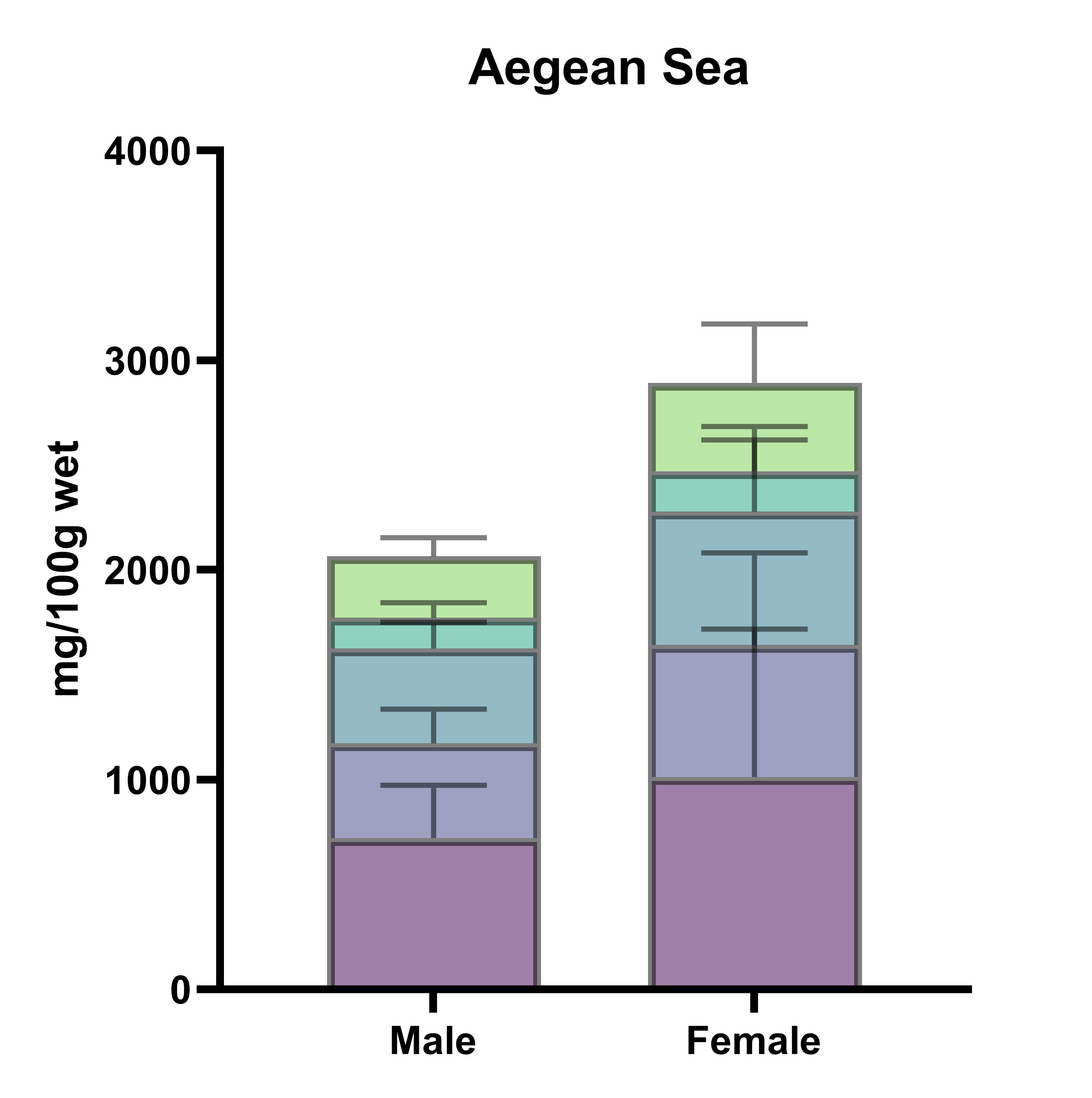

Supplement: Supplementary file 1 [file foods-14-02353-s001.zip › Figure S2a.tif]

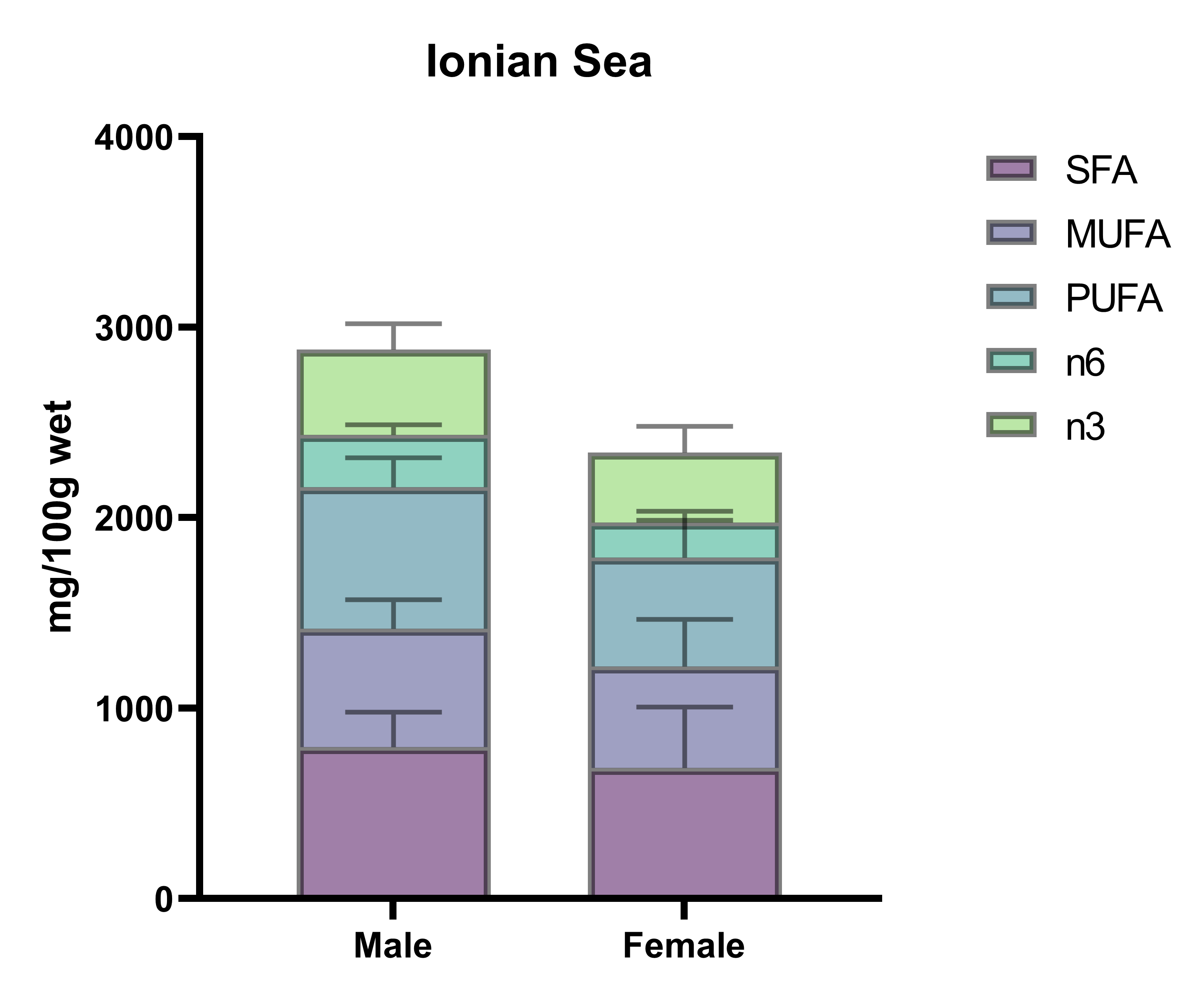

Supplement: Supplementary file 1 [file foods-14-02353-s001.zip › Figure S2b.tif]

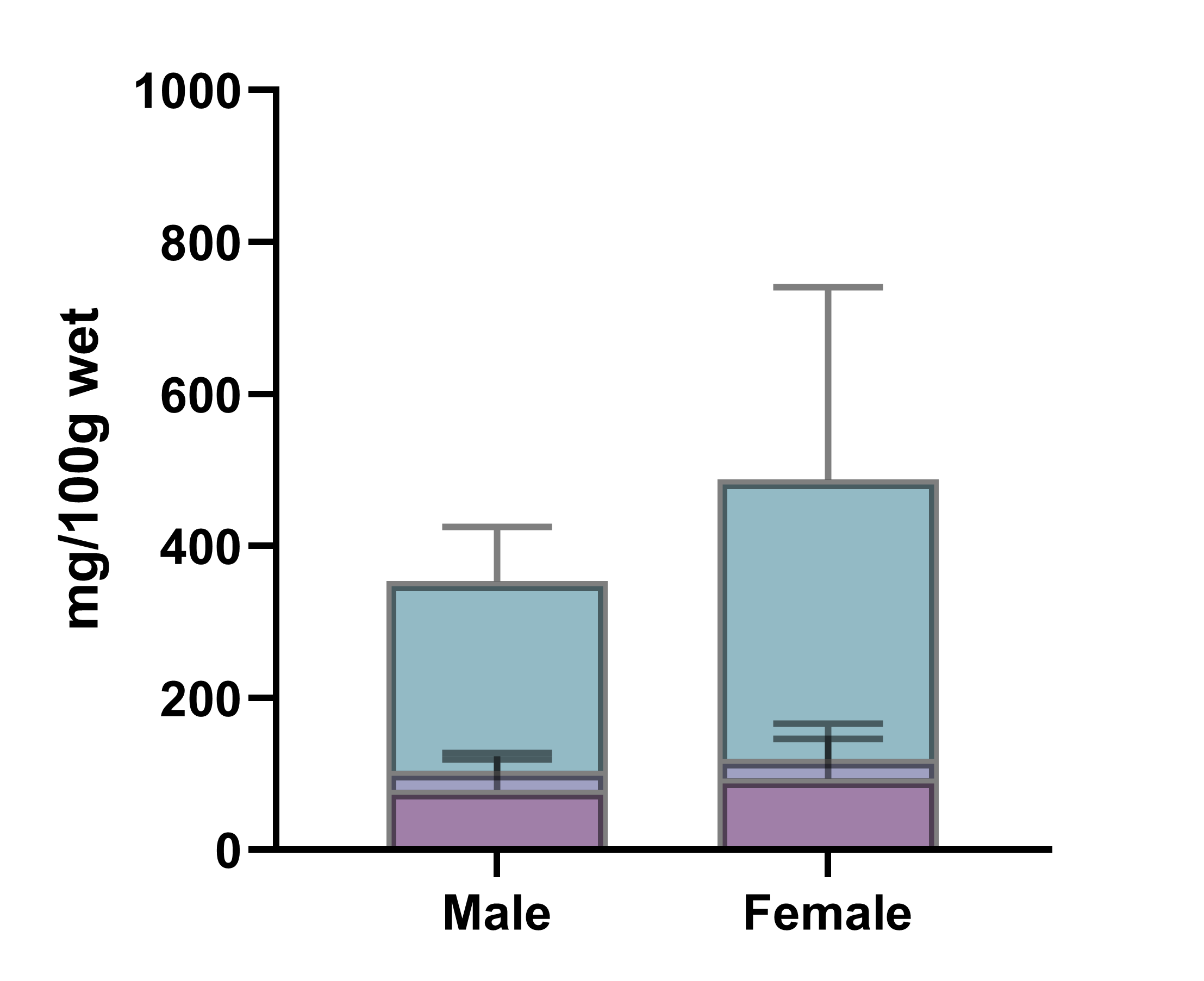

Supplement: Supplementary file 1 [file foods-14-02353-s001.zip › Figure S2c.tif]

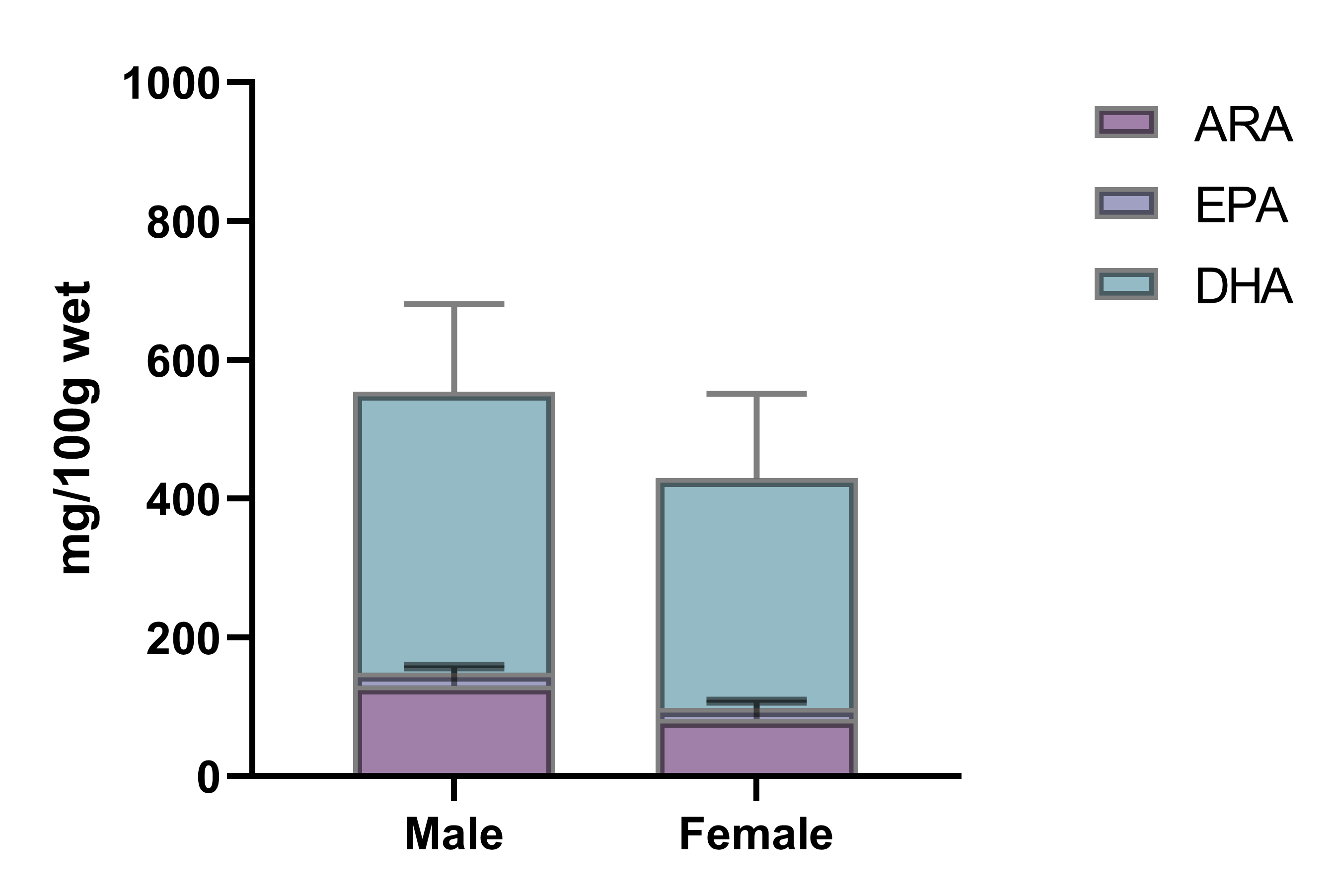

Supplement: Supplementary file 1 [file foods-14-02353-s001.zip › Figure S2d.tif]
